# P-1379. Predictors of Death and Therapeutic Failure in Patients Treated with a Shortened All-Oral Regimen for Multidrug- or Rifampicin-Resistant Tuberculosis (MDR/RR-TB) in Lesotho

**DOI:** 10.1093/ofid/ofaf695.1566

**Published:** 2026-01-11

**Authors:** Edmund Shen, Mikanda Kwabisha Kunda, Stephane Mpinda, Allison LaHood, Letizia Trevisi, Molly Franke, Llang Maama

**Affiliations:** Harvard Medical School, Boston, MA; Partners In Health Lesotho, MASERU, Maseru, Lesotho; Parterns In Health Lesotho, Maseru, Maseru, Lesotho; Harvard T.H. Chan School of Public Health, Boston, Massachusetts; Harvard Medical School, Department of Global Health and Social Medicine, Boston, Massachusetts; Harvard Medical School, Boston, MA; Lesotho Ministry of Health, Maseru, Maseru, Lesotho

## Abstract

**Background:**

MDR/RR-TB remains an important contributor to the global TB disease burden. Historically, effective therapy was hindered by prolonged regimens and drug toxicity. Recent clinical trials and observational studies demonstrated the potential of shortened all-oral regimens in improving treatment outcomes, leading the WHO to recommend several standardized 6 and 9-month regimens. Which patients are at high risk for unfavorable outcomes under these regimens is largely unknown. Here, we report clinical and radiographic predictors of treatment failure for a 9-month all-oral regimen implemented in Lesotho.

**Methods:**

As part of the STEM-TB (NCT05871489) multinational prospective cohort study, we recruited adult participants with bacteriologically confirmed MDR/RR pulmonary TB under operational research conditions. Patients with confirmed fluoroquinolone susceptibility and no drug contraindications were treated with a 9-month regimen containing levofloxacin, bedaquiline, delamanid, linezolid, and clofazimine. We assessed end-of-treatment outcomes and performed logistic regression to evaluate baseline factors that predict death and therapeutic failure.

**Results:**

Of 237 participants included in efficacy analyses, median age was 41 (IQR:31-56), 70 (30%) were female, and 147 (62%) were baseline HIV positive. Clinical cure was achieved in 215 (91%) individuals, and therapeutic failure or TB-related mortality was noted in 22 (9%). Baseline patient features inversely related to treatment success included radiographic evidence of fibrosis (OR: 0.31, 95%CI: 0.12-0.81), and presence of the extensive disease phenotype (cavitary lung disease and smear grade of 2+ or greater) (OR: 0.37, 95%CI: 0.13-1.1). Severe lung damage, defined here as multilobar fibrosis with any degree of concomitant cavitary disease and smear positivity, was also highly predictive of death or therapeutic failure (OR:0.14, 95%CI: 0.04-0.53).

**Conclusion:**

Combinations of baseline radiographic and smear features are predictive of unfavorable outcomes, and may have utility in identifying and stratifying patients at high risk for treatment failure. These findings contribute to the development of personalized management strategies for high-risk patients undergoing shortened all-oral MDR/RR-TB regimens.

**Disclosures:**

All Authors: No reported disclosures

